# Hybrid Hospital-at-Home Program in Singapore: Ethnographic Study

**DOI:** 10.2196/66107

**Published:** 2025-06-02

**Authors:** Stephanie Ko, Daphne Cheong, Shi Yun Low, Shefaly Shorey

**Affiliations:** 1 Division of Advanced Internal Medicine Department of Medicine National University Hospital Singapore Singapore; 2 National University Health System at Home (NUHS@Home) National University Health System Singapore Singapore; 3 Alice Lee Centre for Nursing Studies Yoo Loo Lin School of Medicine National University of Singapore Singapore Singapore

**Keywords:** interview, participant observation, qualitative, ethnography, hospital at home, hybrid hospital, ethnographic study, singapore, patient experience, remote care, healthcare delivery, telehealth, virtual consultations, healthcare technology

## Abstract

**Background:**

Hospital-at-Home (HaH) programs deliver hospital-level care in the home setting, traditionally emphasizing high-touch, in-person clinical visits. In recent years, especially during the COVID-19 pandemic, HaH models have rapidly evolved to incorporate digital technologies, enabling remote consultations and monitoring as part of care delivery. While both in-person and remote HaH modalities have individually shown positive outcomes with patient experience and clinical safety, less is known about how hybrid models, combining both modalities, are experienced by patients and caregivers. This knowledge gap is particularly salient in Asian health care systems, where strong familial caregiving norms and blended influences of Western and traditional medicine may uniquely shape care experiences.

**Objective:**

This study aims to explore the experiences of patients and caregivers receiving acute care through a combination of remote consultations and in-person visits within a hybrid HaH program in Singapore.

**Methods:**

An ethnographic qualitative research design was used to capture real-world experiences of HaH care delivery. Data collection occurred between November 2022 and May 2023 through participant observations of home visits, remote consultations, and semistructured interviews. Data were thematically analyzed using an inductive approach.

**Results:**

The participant observation included data from 25 patients and caregivers. A total of 4 participants were excluded from the in-depth semistructured interviews—3 were uncontactable and one was re-admitted to the hospital, resulting in a final interview sample of 21 participants. A total of three key themes, supported by 12 subthemes, were identified: (1) positive experiences of remote and home visits—participants expressed feelings of comfort, convenience, safety, and a sense of empowerment; (2) patient-provider dynamics—this theme highlighted the importance of in-person visits while noting challenges such as technology usability, complex medical communication, and difficulties in building rapport; and (3) complexities of the home as a care setting—participants reported environmental limitations, hidden financial burdens, the need for advance scheduling, perceived safety risks, and role confusion among caregivers.

**Conclusions:**

This study underscores the multifaceted nature of delivering acute care at home through hybrid HaH models that integrate both remote and in-person visits. Physician home visits are crucial for enhancing patient and caregiver experiences, supporting remote care. Certain patient groups with limited caregiver support, dual caregiving roles, lower digital literacy, or concerns about the feasibility of home care may require more frequent in-person visits. Public education is essential to dispel misconceptions about home-based care and align societal norms around recovery at home. Financing models should also account for hidden costs to promote equitable access. Policy makers and clinicians should consider both operational needs, such as flexible coordination and digital integration, and societal factors such as varying levels of digital literacy and strong family caregiving norms when designing hybrid HaH programs, particularly in culturally diverse settings like Singapore.

## Introduction

Hospital-at-Home (HaH) provides acute hospital-level care to patients in their homes as a substitute for traditional ward-based hospitalization [1]. Originally developed in the 1970s, HaH has since gained acceptance in health care systems across Australia, Europe, and the United States for its potential to alleviate acute care bed shortages, reduce costs, and improve patient outcomes [2-8]. The recent rapid expansion of HaH across the US, the United Kingdom, and Singapore was fueled initially by the COVID-19 pandemic but sustained by ongoing challenges in hospital bed capacity as populations age [9,10].

The conventional high-touch approach, often observed in older HaH pilot studies, tends to offer patients greater reassurance and confidence in care [11,12]. However, a high-tech approach has been adopted in recent waves of HaH expansion, with remote visits emerging as an alternative to home visits for patient assessment, especially during the COVID-19 pandemic [13-15]. A trial comparing home to remote visits showed noninferior outcomes with remote visits, although home visits were still necessary to support many patients receiving remote care [16]. Likewise, a HaH program providing a hybrid of remote and in-person care has demonstrated positive clinical outcomes [17]. This shift positions HaH not only as an alternative care setting but also as a model at the forefront of technology-enabled acute care, aligning with broader digital health trends such as hybrid care delivery and patient-centric remote care ecosystems.

Caregivers often play a central role in supporting patients during HaH admissions, particularly concerning emotional, logistical, and sometimes clinical tasks [18,19]. Similarly, health care providers are key agents in delivering and adapting care within this nontraditional environment, and their interactions with both patients and caregivers shape the overall care experience [20]. For example, nurses who often deliver in-person and remote components of HaH care play an essential role in navigating this transition to coordinate care and maintain patient safety [6,21]. As HaH care involves ongoing collaboration among patients, informal caregivers, and health care providers, understanding the interplay between them is crucial to capture the full scope of care experiences. Especially, patient and caregiver perceptions of this hybrid model of care remain underexplored, particularly within Asian contexts. Unlike Western settings where older adults are more likely to live alone, many older adults in Asia reside with family members, often relying on caregivers for daily support [22]. This distinction may influence how caregivers perceive and navigate HaH care in an Asian context, such as Singapore [23]. As such, gaining insights into their experiences is essential for refining future HaH programs. This understanding can facilitate optimizing resource utilization, improving patient and caregiver experience, and contributing to better clinical outcomes.

While HaH has been explored through a phenomenological lens, offering valuable insights into the lived experiences of patients and caregivers in the United Kingdom, these findings are inherently shaped by their cultural and health care context [24]. In contrast, Singapore’s distinctive integration of traditional and Western medical practices, strong family caregiving norms, and unique health care delivery model may shape patient and caregiver experiences differently [25-27]. Hence, understanding these cultural and systemic nuances is essential for tailoring HaH programs to Singapore’s diverse population. Adopting an ethnographic approach allows for a complementary perspective by capturing how care relationships, decision-making processes, and perceptions of home-based acute care emerge in a real-world environment. To address this gap, the present study aims to explore the experiences of patients and caregivers receiving acute care in their natural environments through remote and home visits within a HaH program in Singapore.

## Methods

### Study Design

An ethnographic qualitative research design was used to explore experiences encountered by patients and caregivers in a HaH program. Different sources (ie, observations, field notes, and interviews with patients and caregivers) were used to triangulate data and provide a corroborated understanding of the experience of HaH within the Singapore context, enhancing the dependability of this study’s findings [28]. The Standards for Reporting Qualitative Research (SRQR) checklist guided the reporting of this qualitative study [29] (Multimedia Appendix 1).

### Setting

The National University Health System is an academic health care system in the western region of Singapore, consisting of 3 acute care hospitals. The National University Health System at Home (NUHS@Home) program was established in 2020 to provide HaH services for patients who would otherwise require inpatient admission to an acute care ward. Patients admitted to HaH are referred directly from emergency departments or hospital inpatient teams, and all admissions meet clinical criteria for acute-level care. Admission to NUHS@Home is recognized as a formal hospital admission, enabling Singapore residents to access government subsidies to help offset care costs. Specifically, patients can use MediSave and MediShield Life for coverage. MediSave is a compulsory national medical savings scheme that requires working Singaporeans and Permanent Residents to set aside a portion of their income into a dedicated savings account to pay for future hospitalization, day surgery, and certain outpatient expenses. MediShield Life, on the other hand, is a basic, universal health insurance plan administered by the Central Provident Fund (CPF) Board. It provides lifelong protection against large hospital bills and selected costly outpatient treatments, regardless of age or preexisting conditions. MediShield Life premiums can be paid using funds from the individual's MediSave account [30]. Together, these schemes help ensure that patients receiving care under NUHS@Home are financially supported in a manner like traditional hospital-based admissions. Eligibility for NUHS@Home was determined based on clinical stability and the feasibility of providing safe hospital-level care at home, with final admission decisions made collaboratively by the designated HaH clinical team and the referring physicians. Patients enrolled in NUHS@Home received four key components of care: (1) daily remote visits by physicians followed by home visits if a physical examination was necessary, (2) nursing reviews for caregiver training on specific procedures such as draining urinary catheter bag, (3) remote vital signs monitoring, and (4) engagement of allied health visits or transport to hospital for imaging as required. Collectively, these components replicate the intensity of medical supervision, timely monitoring, and multidisciplinary coordination typically found in acute inpatient settings. During the study period, procedural tasks such as venipuncture and intravenous therapy were primarily performed by third-party agency nurses while hospital-based nurses focused on comprehensive clinical care such as patient physical assessments, caregiver education, and training. These operational workflows of NUHS@Home replicate the traditional inpatient acute care that patients typically receive from hospitals [14].

### Sampling and Eligibility

Purposive sampling was used to recruit eligible participants who met the following criteria: (1) patients and caregivers aged 21 years and older and (2) being admitted to NUHS@Home. Participants were excluded if they were uncontactable or if their clinical condition deteriorated, requiring readmission to the hospital for closer monitoring.

### Data Collection

Data were collected via observations of patients, caregivers, and health care providers during home visits in patients’ homes and remote consultations conducted in the HaH office between November 2022 and May 2023. Follow-up individual interviews with patients or their caregivers (if patients were unable to communicate in English) were also conducted. One of the trained research team members (DC), who was an experienced nurse, collected all the data. The researcher received training from the senior research team member (SS) on conducting interviews and meaningful observations without intervening in the care process. A female research assistant and registered nurse (DC) with a BSc in Nursing, who had no direct relationship with the HaH program, observed both home visits and remote consultations by attending these sessions physically and documenting field notes using an observation grid that was adapted from the Total Quality Framework of Schaefer et al [31]. A pilot observation was conducted with 1 participant, and the observation grid was determined by the study team to effectively capture the nuances and interactions among the health care providers, patients, and caregivers that were of interest to this study. No changes were made to the observation grid after the pilot testing. The data from the pilot observation was included in the final analysis as it contributed valuable data to the study. A total of 25 participant observations were conducted, comprising both in-person and remote consultations. During observations, the researcher (DC), a registered nurse, did not wear a uniform like other health care providers and was embedded within the HaH team as a nonclinical observer. To facilitate access and build rapport with the participants, the researcher was introduced as a nonclinical member of the care team conducting a study to explore patients’ and providers’ experiences with the HaH model. However, it was made explicitly clear to patients, caregivers, and staff that the researcher was not involved in any clinical decision-making and was present solely for observation and data collection. This dual identity was carefully managed through transparent communication, verbal clarification during initial interactions, and written informed consent procedures. As 1-2 clinical team members visited the patient’s home at each time point, no observer bias was reported by either the study participants or the researcher. Clinical encounters during home visits were multifaceted, involving diagnostic assessments, procedural interventions, and logistical arrangements, resulting in the researcher’s presence being quickly overlooked by both the clinical team and the participants. Field notes were written in full during and immediately after each observation to avoid missing out on any pertinent information.

Following the observations, interviews with patients or caregivers were conducted either in person or on the web, based on their preferences. The interview guide was developed based on the literature [32,33] and the clinical and methodological expertise of the research team members (SS and SK). The same trained female research assistant (DC) who collected the study data also conducted a pilot interview with one participant to evaluate the flow and relevance of the interview guide, ensuring alignment with the research objectives. No further modifications were needed, and the data from the pilot interview was included in the final analysis as it contributed valuable insights to the study. All interviews were conducted face-to-face in English with a total of 21 participants. A total of 4 participants were not interviewed due to various reasons (3 uncontactable and 1 who got readmitted to the hospital). However, observational data from home or remote visits involving these 4 participants were still included in the overall analysis as they contributed valuable insights into the experiences of patients and caregivers. Each interview was audio-recorded and transcribed verbatim. At the end of each interview, a verbal summary of key points was provided for participants to seek clarification and make any necessary amendments to their responses. Data saturation was reached after the 19th interview, as no new concepts emerged [34]. Additional 2 interviews were conducted to confirm data saturation. The observation grid and interview guide are provided in Multimedia Appendix 2.

### Rigor

Guba and Lincoln’s [35] guidelines were used to establish trustworthiness. Credibility was maintained through prolonged engagement and member-checking with participants during data collection, as well as investigator triangulation during data analysis. Transferability was established by providing rich, thick descriptions of participants in the HaH program, supported by direct quotes. Dependability was established by keeping a detailed audit trail that included interview transcripts, coding, and the analytical process. Confirmability was enhanced using a reflexive journal throughout the research process. Reflexivity, as emphasized by Teh and Lek [36], is a critical aspect of qualitative research that minimizes the influence of unconscious researcher biases during data interpretation. The journal provided a transparent account of these reflections, further strengthening the study’s trustworthiness [37].

### Data Analysis

Data analysis occurred concurrently with data collection, following a 3-step thematic approach informed by Braun and Clarke’s [38] and Creswell and Poth’s [39] frameworks for thematic analysis and aligned with an ethnographic research design. Drawing on principles of symbolic interactionism which emphasizes the meaning-making processes within social interactions, the analysis sought to capture participants' lived experiences within their natural environments [40]. First, data from field notes and transcribed interviews were independently deconstructed into first-order concepts by 2 independent reviewers (DC and SYL). Initial codes were developed by identifying meaningful units of information and relevant social or cultural concepts embedded in the participants' narratives. Second, the coded data were systematically organized into preliminary themes, capturing patterns of behavior, interaction, and meaning within the home care context. Researchers paid attention to the symbolic significance of participant actions and words, consistent with ethnographic inquiry principles [41]. Finally, emerging themes were reviewed, refined, and synthesized through iterative discussions among the research team to conceptualize the central phenomenon into a coherent descriptive narrative (Multimedia Appendix 2). Discrepancies between reviewers were addressed through dialogue until consensus was achieved, ensuring analytic rigor and reflexivity throughout the process.

### Positionality Statement

The research team comprised an internal medicine clinician, 2 registered nurses, and a public health researcher with expertise in qualitative methodology. This interdisciplinary composition provided diverse perspectives on the HaH model and informed the approach to ethnographic fieldwork. The team’s clinical, methodological, and public health backgrounds enabled them to engage meaningfully with participants and interpret care practices with contextual sensitivity, while also facilitating rapport within the HaH setting. The team recognized that their prior experiences in health care could influence how they observed and interpreted the data. To remain critically aware of their positionality, a reflexive journal was maintained throughout the research process, complemented by regular team meetings. The researchers demonstrated a strong commitment to personal reflexivity by explicitly considering how their professional perspectives could be integrated constructively [42]. This practice helped ensure that the analysis remained grounded in the participants’ narratives, prioritizing the authenticity of their voices [43]. In addition, the research team’s shared background as Singaporeans provided deeper contextual insights into Singapore’s health care delivery system, further enhancing the relevance, sensitivity, and cultural appropriateness of the study’s interpretations.

### Ethics Approval

The study received ethical approval from the National Health Group Domain Specific Review Board (Reference Number: 2020/00345), which serves as the ethics board of the participating institution. The research assistant (DC) approached eligible participants and provided them with detailed information sheets outlining the study’s aims and roles. Written informed consent was obtained from patients, caregivers, and health care providers by the research assistant (DC). Caregivers or Legally Appointed Representatives (LARs) were alternatively approached if the patient could not provide informed consent. Voluntary participation was reinforced. To ensure confidentiality and anonymity, participants’ data were coded using unique serial numbers from 1 to 21. Participants were reimbursed with a token of appreciation of SGD 20 (US $15.55) for sharing their valuable insights.

## Results

### Overview

A total of 25 participant observations were conducted, comprising both in-person visits and remote consultations. In-person observations ranged 30-132 (mean 50.6, SD 28.3) minutes while remote consultations ranged 2-14 (mean 6, SD 3.6) minutes. A total of 21 interviews were conducted with durations ranging 21-64 (mean 33.6, SD 9.6) minutes. Participants were predominantly females (n=13), with diverse ethnic backgrounds, Chinese (n=10), Malay (n=7), and Indian (n=4). The average participant age was 56.7 years. The demographic profiles of the 21 participants are listed in Multimedia Appendix 3.

Three main themes were identified: (1) positive experiences of remote and home visits in HaH, (2) patient-provider dynamics in remote and home visits, and (3) complexities of the home environment as a site of care. These themes were further supported by 12 subthemes (Multimedia Appendix 4).

### Theme 1: Positive Experiences of Remote and Home Visits in HaH

#### Subtheme 1.1: Feelings of Comfort, Convenience, and Safety

The majority of patients expressed comfort in recuperating at home, enjoying home-cooked meals, and appreciating the peace and quiet compared with the potential disturbances experienced in hospitals from other patients or nurses. In addition, they viewed HaH as a safe and time-saving measure for visiting relatives, eliminating the need for travel to hospitals, which they perceived as “ridden with germs” [Patient 24, Interview].

I am happy … this [HaH care] is convenient [for her family members] …my husband is sick…lucky no need to visit me [in the hospital] and I can be with him

Patient 2, Interview

#### Subtheme 1.2: Empowerment of Patients

Many patients demonstrated confidence and took charge of their health by facilitating care procedures at home. For instance, a patient was observed guiding nurses on an intravenous (IV) drip placement based on previous home visit experiences. This was particularly valuable for independent patients who offered insights into how care can be provided at home to minimize impact on their daily living activities.

Patients also considered self-monitoring of vital signs and remote consultations to be “empowering.” Despite observed “hesitance among some patients” [Patient 24, Virtual consultation] to speak up during remote consultations, the majority provided feedback and showed their medications to the doctor over the video call [Patient 7, Virtual consultation] to ensure they received proper care at home.

Patient 11 took the effort to walk across the room to show his ability to take care of himself and adjusted the camera to show the living room to the doctor.

Virtual consultation observation

#### Subtheme 1.3: Empowerment of Caregivers

Likewise, caregivers actively participated in discussions, providing new information and asking questions about patients’ conditions during both home visits and remote consultations. This high level of engagement from caregivers further emphasized their integral role in potentially impacting patient outcomes within HaH.

Daughter of patient 10 was noted as providing information about the different antibiotic regime, etc. (…), proactively raised concerns about rashes and showed them to the doctor.

Home visit observation

### Theme 2: Patient-Provider Dynamics in Remote and Home Visits

#### Subtheme 2:1. Importance of Home Visits

Most patients expressed appreciation for home visits by health care providers during their HaH admission. As Patient 15 aptly stated during an interview, “nothing beats 1-2 physical (…) checking up.” Patients considered physical visits from doctors sufficient to instill a sense of security and assurance for the HaH program.

Home visits make me feel less Kan Cheong (colloquial for anxious), …ya I want them definitely

Patient 11, Interview

#### Subtheme 2:2. Challenges of Operating Technology for Remote Visits

Health care providers depended on patients or their caregivers to operate electronic devices and adjust camera angles for proper assessment of wounds or other areas of interest during remote consultations. However, older patients and caregivers, particularly those living alone, were observed to have difficulty panning the camera effectively, consequently affecting the clinical evaluation.

Healthcare providers seem frustrated due to the poor resolution of the camera used. Ask the patient to use the phone to take photos of the medication.

Patient 20 Virtual Observation

#### Subtheme 2:3. Challenges in Complex Communication in Remote Visits

Disseminating complex instructions appeared more challenging during remote visits compared to home visits. Patients seemed more inclined to discuss care issues and showed clearer understanding when instructions were communicated in person rather than via video call. Many patients expressed confusion regarding available subsidies for HaH, despite receiving calls regarding financial consulting. This highlights potential inadequacies in conducting admissions and discharge processes over remote consultations, as it may not provide patients with sufficient information or reassurance.

All I remember is they told me that this one [HaH care] is 40% cheaper than staying in the ward, but now I don’t know what the bill is like…I worried if I hear properly.

Patient 6, Interview

#### Subtheme 2:4. Barriers and Facilitators to Rapport Building

Continuity of care providers over time, as well as shared demographic backgrounds or interests, was important in fostering strong rapport between patients and health care providers. Patients' behavior varied depending on whether care was provided by vendor nurses or hospital health care providers. Patients interacting with vendor nurses tended to exhibit a “casual” and “laidback” demeanor [Patient 12, Home observation], while patients visited by hospital personnel appeared more “formal/tense” and “attentive” [Patient 5, Home observation].

Patient 12 and his [vendor] nurse observed as smiling and laughing lightly/chuckling in response to nurse’s jokes and seemed to have a friendly relationship. This was not evident among all the patient-nurse dyads.

Home visit observation

Rapport building with patients was notably challenging during virtual consultations with language barriers. In such cases, health care providers often depended on caregivers for translation. This challenge was exemplified when health care providers directed their focus on explaining treatment plans to caregivers instead of patients, potentially causing patients to feel excluded from discussions about their own care.

The caregiver and the doctor had a direct conversation and patient 23 appeared to be excluded from the discussion

Virtual consultation observation

### Theme 3: Complexities of the Home Environment as a Site of Care

#### Subtheme 3:1. Home Environment

The suitability of home environments for treatment varied among patients in the HaH program. While most homes could accommodate basic IV therapy, some were not conducive for health care providers to operate in. Health care providers were observed to have difficulties working effectively in homes that were too cluttered, narrow, or dimly lit.

Nurses were seen squinting due to dim lighting and shadow, using their phone lights while trying to change their patient’s dressing, and squeezing past (…) a narrow gap by patient 10’s bedside table while trying to access to the dressing site.

Home visit observation

#### Subtheme 3:2. Needing Advanced Notice for Home Visits

Patients expressed a preference for receiving advanced notice and accurate timing of home visits to avoid feeling caught off guard. For example, during a home visit, a patient requested for additional time to change into her headscarf for religious modesty despite the nurse already being present for the home visit. Patients also expressed frustration over disrupted rest due to uncertainty about the timing of health care providers’ home visits.

I didn’t know what time they are coming … the last one they came right, I was actually asleep. I was more like shocked.

Patient 22, Interview

#### Subtheme 3:3. Perceived Risks of the Home Environment

The constrained environment and the absence of specialized infection control measures in the home setting raised concerns among some patients regarding the capacity to adequately contain the spread of infections at home.

In a hospital environment there are infection control measures…at home nothing…this is worrying

Patient 9, Interview

In addition, concerns were raised regarding the system of medication delivery within the HaH program. Some patients expressed concerns about risks to privacy and medication safety within the current arrangements that involve having medication left unattended for collection at the doorstep of their home.

The last IV drip they sent over. It’s a third-party issue la ... Basically, they just left it outside the house … my name, my name is there.

Patient 3, Interview

#### Subtheme 3:4. Hidden Costs

Patients reported incurring “extra cost” due to being admitted to HaH, which might not have arisen if admitted to an inpatient hospital ward. Several patients had to stop preparing their meals at homes and relied on takeaways, food deliveries, or purchased frozen meals that were more expensive. Moreover, patients expressed that their caregivers often felt the need to take leave from work or arrange to work from home to remain at home to care for them due to “safety concerns” [Patient 8, Interview].

[Transition to HaH care] so busy… so in the end, I mostly bought all the soup-based [food].

Patient 11, Interview

#### Subtheme 3:5. Role Confusion

Some female patients reported difficulties balancing their roles within the household or workplace while receiving HaH care. While HaH offered the comfort of home recuperation, it inadvertently complicated traditional caregiving roles for women who also assumed responsibilities as housewives, mothers, or daughters. One patient expressed frustration with her family's inability to understand her need for recovery, while others felt obligated to resume normal household duties despite their condition.

In [the] hospital definitely the nurse [will not] allow [the patient to do cooking or other household chores] right, but in [the] house ah you [are by] yourself ah so your family say, oh okay she can do everything what, what’s the problem.

Patient 18, Interview

Many patients found it challenging to communicate the seriousness of their medical condition to employers, resulting in impeded recovery. Despite being advised to rest, patients felt compelled to resume working or were expected to continue working from home. This highlights that societal and work norms may not yet fully accommodate the unique demands of the HaH model of care.

Unofficially, unofficially, I work from home already…bo pian [no choice]...

Patient 19, Interview

## Discussion

### Principal Findings

This study explored patient and caregiver experiences within a hybrid HaH program in Singapore, yielding insights into the multifaceted nature of home-based care. Participants reported feelings of comfort, convenience, and safety associated with receiving care in the home alongside a sense of empowerment fostered in both patients and caregivers. Nonetheless, several challenges were identified relating to the use of technology for remote visits, difficulties with complex communication, and the establishment of rapport. In contrast, the continued presence of home visits was perceived as essential when scheduled with adequate advance notice. In addition, environmental concerns, hidden costs and role ambiguity further underscore the importance of contextually attuned and flexible care models when implementing HaH services in culturally diverse settings.

The patients in this study expressed that receiving medical treatment at home offered them reassurance and a sense of comfort in their familiar surroundings. Moreover, this service also provides a safer, more hygienic environment and enhances time efficiency for family visits. This aligns with existing literature, with studies conducted in the United Kingdom and Denmark suggesting that patients reported positive experiences with the HaH care model [32,42]. They attribute this to the comfort of receiving treatment at home and the added convenience of spending time with friends and family, as it helps overcome challenges such as restrictive visiting hours. Hence, these findings underscore the importance of incorporating patient preferences into home care delivery, reinforcing that high-quality care can be provided while promoting well-being and family engagement.

A key enabler for successful patient engagement in both home and remote visits was the proactiveness among patients and caregivers. This aligns with known associations between patient activation and positive health-related outcomes [43], suggesting enhanced compliance or communication with health care providers [44]. The observed proactiveness is therefore not merely a behavioral aspect but potentially a contributory factor to improved care outcomes, reinforcing patients' empowerment and knowledge base in managing their health care [45]. The role of caregivers, already critical stakeholders in HaH care [19,46], becomes even more pronounced when remote visits are planned. Beyond direct caregiving tasks, caregivers often serve as vital communication links between patients and health care providers, contributing to care coordination and continuity [47]. Therefore, their role should be recognized as a critical dimension in HaH care, meriting specific attention to policy and operational guidelines. Importantly, an assessment of patient and caregiver proactiveness may help identify patients who could benefit more from frequent home visits.

While patients recognized the potential advantages of the HaH care model, such as remote monitoring and teleconsultations, some felt that in-person home visits by health care providers offered an added sense of reassurance, helping to further allay their anxiety. This is consistent with a study conducted in the United Kingdom, which suggests that patients valued daily visits from the HaH team and these interactions provided them with confidence, and a sense of security if medical attention was warranted [32]. However, one of the key strengths of the HaH care model lies in its flexibility to adopt a hybrid approach that integrates both teleconsultations and physical home visits when necessary. Relying solely on daily in-person visits to alleviate patient anxiety may lead to increased hidden health care costs and inefficient use of resources, such as prolonged travel time for health care providers [48,49]. This highlights a critical need to balance patient preferences with optimal resource usage. Therefore, future HaH programs could focus on enhancing patient education, and empowering individuals with the knowledge and confidence to respond appropriately in the event of clinical deterioration while maintaining efficient and sustainable use of health care resources.

Patients identified several challenges, such as limited digital literacy, complexities in communicating medical decisions, and difficulties in building rapport with health care providers. This aligns with existing literature, with studies conducted in the United States and Sweden, where patients similarly expressed concerns about technology unfamiliarity [50,51]. Furthermore, the struggle to communicate intricate medical information and build meaningful connections with health care providers, potentially due to the lack of face-to-face interactions, is consistent with existing literature [52]. A unique finding in this study was the influence of attire on patient interactions. Vendor nurses without formal uniforms fostered a less hierarchical relationship with patients, in contrast to interactions involving uniformed hospital personnel with whom patients expressed more formality. While this may stem from respect for health care professionals and authority figures in Asian cultures, this deference may affect communication patterns, inhibiting or facilitating the exchange of information depending on the context [1,53-55]. Considering attire as a nonverbal cue can either reinforce or diminish traditional hierarchies in health care is crucial for optimizing patient comfort and engagement in HaH. Although the HaH care model offers numerous benefits, it also presents certain drawbacks that must be carefully considered to ensure safer care for patients. Future studies could explore digital literacy interventions aimed at empowering patients to navigate telehealth platforms confidently while examining communication strategies that effectively convey complex medical information in remote settings while preserving patient-provider relationships.

Patients in this study shared concerns about their home environments, highlighting that they were often unsuitable for treatments such as intravenous antibiotics and could pose a higher risk of infection due to inadequate infection control measures. In addition, they voiced discomfort with medications being left unattended outside their homes, which they felt compromised their privacy. These findings align with existing literature from Australia and the United Kingdom, suggesting the crucial role of home environments in shaping patient recovery and care experiences, ultimately fostering a more holistic and patient-centered recovery process compared to conventional inpatient care [56,57]. Future studies should consider integrating environmental risk assessments into the patient triage process to better identify those who are most suitable for the hybrid HaH care model. Developing strategies to enhance infection control and secure medication delivery methods is warranted to improve patient safety and privacy without undermining the convenience offered by HaH care.

Patients in this study also highlighted a preference for scheduled home visit timings to avoid disruptions to their rest and daily routine. In addition, they noted that perceived cost savings with HaH care were often overestimated due to hidden expenses such as frequent food deliveries and caregivers having to take time off work to provide support, which contributed to the overall burden of home-based care. This is congruent with studies conducted in the United Kingdom and Australia, where patients voiced similar concerns about scheduling home visit timings and underestimating cost savings [56,58]. Future research should explore patient-centered scheduling models that align with individual routines, which may enhance care adherence and clinical outcomes. Moreover, studies should aim to quantify hidden and indirect costs associated with HaH care from both patient and caregiver perspectives. Investigating the extent of caregiver burden in this context can also inform the development of supportive mechanisms such as paid caregiver support or subsidized services to alleviate caregiver strain. Stakeholders should consider implementing caregiver leave policies and expanding financial assistance schemes to ensure that the shift to HaH care does not disproportionately place the burden on informal caregivers.

The patients in our study reported difficulties in balancing familial and workplace responsibilities while receiving HaH care. Many experienced conflicts stemming from traditional gender roles, such as managing household duties, which hindered their ability to fully focus on recovery. Interestingly, this contrasts with findings from a study conducted in Norway, where family caregivers actively supported the patient and ensured they had adequate rest throughout the HaH admission. This may be influenced by cultural factors such as multigenerational Asian household dynamics and Confucian principles of “filial piety,” which often place caregiving responsibilities on daughters, alongside broader cultural norms that assign women the role of primary caretakers [19]. These findings suggest the importance of incorporating culturally sensitive care planning into HaH models to identify potential role conflicts early in the admission process. Promoting shared caregiving responsibilities within the household may help redistribute duties and support patient recovery. Future studies could explore the influence of cultural norms and gender expectations on patient experiences and recovery in HaH settings to inform the development of tailored patient and family education that supports equitable care practices.

### Limitations

This study has several limitations. First, the ethnographic methodology, while rich in qualitative detail, may have introduced observer bias, particularly among health care providers aware that the researcher is collecting observational data. To mitigate this, reflexive journaling and investigator triangulation were maintained throughout the research process to enhance self-awareness and critically examine potential bias. While these strategies enhanced the study’s trustworthiness, the influence of researcher subjectivity cannot be eliminated and remains a limitation of the study. Second, the study was limited by triangulating observations with a single set of patient interviews. Future research could incorporate the perspectives of health care providers and caregivers. Thirdly, as observations and interviews did not address patient experiences in traditional hospital wards, hidden costs and experiences cannot be compared to alternative methods for hospitalization. Moreover, demographic data for caregivers and health care providers were not collected, which may have limited the ability to contextualize how factors such as age, caregiving role, or professional background may have influenced the patients' experience. Future research should consider capturing such information to better interpret the diversity of experiences and enhance the transferability of findings across different settings. In addition, the sample was largely composed of Chinese participants with smaller representations from Malay and Indian participants. Although this reflects the major ethnic composition of Singapore, future research should strive for a more representative sample distribution to better capture diverse perspectives and broader cultural trends. Finally, different socio-economic groups were not purposively sampled. Future research should delve deeper into the varied experiences of HaH across socio-economic groups, particularly as policy discussions around financing this model of care evolve.

### Implications for Future Research and Practice

This study highlights the importance of comfort, convenience, and perceived safety as key contributors to patient satisfaction and engagement in HaH care. These elements, rooted in the familiar home environment, supported sustained participation and positively influenced care outcomes. Patient empowerment emerged as a key theme with individuals taking a more active role in managing their care, signaling a shift toward greater autonomy. Similarly, caregivers are evolving into essential partners in home-based acute care. To support this, stakeholders should implement structured caregiver training, offer financial and emotional support mechanisms, and incorporate digital health literacy into routine HaH care delivery.

The continued importance of physician home visits highlights the need to determine optimal visit frequency and establish patient selection criteria that balance reassurance with efficient resource use. With patients reporting challenges with technology usability, communication of complex medical issues, and rapport building within HaH care, stakeholders should consider developing user-friendly, multilingual telehealth platforms and clinician training in remote communication techniques. In addition, implementing strategies to support relational continuity, such as assigning consistent care teams, may enhance patient-provider interactions and strengthen trust in remote care delivery.

Patients’ concerns about the suitability and safety of their homes for acute care, such as infection control limitations and space constraints, highlight the need for structured home readiness assessments as part of the HaH triaging process. Furthermore, hidden costs such as increased adoption of food delivery services, caregiver time-off, and role confusion between patients and caregivers underscore the importance of factoring in financial and familial dimensions when considering the sustainability and equity of HaH programs. Future research should explore how these contextual factors affect patient and caregiver outcomes, particularly within multigenerational households in Singapore.

### Conclusion

Our study highlights the complexities of providing acute care at home through a combination of remote and home visits. Our findings support the strategic use of doctor home visits as a core component of HaH programs, which are essential to optimize both patient and caregiver experience and the effectiveness of subsequent remote visits. Beyond clinical necessity, patients with the following features may benefit from more frequent home visits: (1) lower levels of proactiveness, (2) less engaged caregivers or living alone, (3) having secondary roles as caregivers, (4) lower technological literacy, and (5) having anxiety over the appropriateness of home as a care setting. Societal education can help address: (1) perceptions of risks of home versus hospital wards, (2) role conflicts experienced by patients who are also caregivers, and (3) expectations of employers when patients are hospitalized at home. Financing models of HaH should incorporate “hidden” costs of HaH, such as food provision or additional caregiving support, to ensure equity across a broader population spectrum.

## Appendix

Multimedia Appendix 1 SRQR (Standards for Reporting Qualitative Research) checklist.

Multimedia Appendix 2 Observation grid (home and virtual observations).

Multimedia Appendix 3 Demographic profile of the participants.

Multimedia Appendix 4 Key themes and subthemes.

## Abbreviations

CPF: Central Provident Fund
HaH: Hospital-at-Home
IV: intravenous
LAR: legally appointed representative
NUHS@Home: National University Health System at Home
SRQR: Standards for Reporting Qualitative Research checklist

## Data Availability

The datasets generated or analyzed during this study are not publicly available due to privacy but are available from the corresponding author on reasonable request.
