# Supplementary material for: Hybrid Hospital-at-Home Program in Singapore: Ethnographic Study
Source: J Med Internet Res. 2025 Jun 2;27:e66107. doi: 10.2196/66107 (PMC12171641; doi:10.2196/66107)
Supplement: Multimedia Appendix 2 [file jmir_v27i1e66107_app2.docx]

**Appendix 2:** Observation Grid (Home and virtual observations)

**Bed 1: Home observations**

| Patient identification code and diagnosis: | Current length of admission (days): | | |  | Date: | Start time: | | Stop time: | |
| --- | --- | --- | --- | --- | --- | --- | --- | --- | --- |
|  | People present: | | | | | | | | |
| Area of Observation |  | Patient | | | | | Caregiver: | | Care provider: |
| Home environment/ atmosphere |  | | | | | |  | |  |
| Sequence of events |  | | | | | |  | |  |
| Area of Observation |  | | Patient | | | | Caregiver: | | Care provider: |
| Context (What else is going on?) |  | | | | | |  | |  |
| Interactions (What, by whom, where) |  | | | | | |  | |  |
| General mood (What, how conveyed, by whom) |  | | | | | |  | |  |
| Other areas of observation |  | | | | | |  | |  |
| Reflexive comments |  | | | | | | | | |

Fieldnotes – other/unstructured observations

**Appendix 2.** Snippet of Interview Guide on Perspectives towards the Hospital-at-Home model of care (Questions were modified based on participants’ responses)

**Research question:** What are the perspectives, barriers, and facilitators of patients and caregivers receiving the Hospital-at-Home model of care?

| Introduction | 1. Introduce self and aim of the study |
| --- | --- |
| Guiding questions (Patient version) | 1. What was your experience of NUHS@Home like? 2. How did you find the treatment process at home? 3. What, if any, are the advantages and benefits of being admitted to NUHS@Home? 4. What, if any, are the challenges and difficulties of being admitted to NUHS@Home? 5. What is the most important thing about healthcare to you? 6. Did this program meet your expectations? Why or why not? 7. Would you choose to be admitted to NUHS@Home if they needed hospitalisation in the future? Why or why not? 8. How did you feel about vital signs monitoring system at home? 9. What is your experience of video calls from the doctor during hospital at home? 10. What, if any, is your experience with doctors or nurses not from NUH that attended to you during your stay? 11. What improvements, if any, can be made to the NUHS@Home programme? |
| Guiding questions (caregiver version) | 1. Why did they choose for enrolment into this study? 2. What was the experience of NUHS@Home like? 3. What, if any, are the advantages and benefits of being admitted to NUHS@Home? 4. What, if any, are the challenges and difficulties of being admitted to NUHS@Home? 5. Did this program meet your expectations? Why or why not? 6. Would you choose Home Hospital again if your loved ones needed hospitalisation in the future? Why or why not? 7. How did you feel about continuous vital signs monitoring on your loved ones at home? 8. What, if any, is your experience of video calls from the doctor during hospital at home? 9. What, if any, is your experience with doctors or nurses not from NUH that attended to your loved ones during your stay? 10. What improvements, if any, can be made to the NUHS@Home programme? |

**Appendix 2:** Example quotes from observation notes and interviews

| **Theme** | **Sub-theme** | **Data Source** | **Example Quotes/ Field Notes** |
| --- | --- | --- | --- |
| Positive experiences of remote and home visits in HaH | Feelings of comfort, convenience, and safety | Interview | *“In hospital, the food wise is really very (laughs) not nice; My mother-in-law cooks so, at least it's like maybe I'm also used to her food… it’s nice to get comfort food…”* – Patient 2 |
|  |  | Interview | *“I am happy … this [HaH programme] is convenient [for her family members] …my husband is sick…lucky no need to visit me [in the hospital]”* – Patient 2 |
|  | Empowerment of patients | Home visit observation | Patient 18 took ownership of having a suitable continuity of care among healthcare providers for her and asked the nurse to raise the IV drip as per what the previous nurse had done. |
|  |  | Home visit observation | Patient 1 encouraged a nurse to step on his bed to reach the curtain rail where the IV drip is hung so that there are no issues with the IV drip flow. |
|  |  | Interview | *“For IV plug … that one hurt quite a bit and I couldn’t shower. Uh, I also walking around, moving around with just using one hand; there was a lot of mobility issues because I wasn't able to use my hand at all; going to the toilet. Simple things like brushing my teeth, opening my food, opening my water bottle, washing my face…so I decided to request for the change [of cannulation site]…”* – Patient 3 |
|  |  | Virtual consultation observation | Patient 11 took the effort to walk across the room to show his ability to take care of himself and adjusted the camera to show the living room to the doctor. |
|  |  | Interview | “*It was too big for me, so I told them [healthcare providers] I am not comfortable to use it and give me easy machine…* *and it was replaced*” – Patient 20 |
|  | Empowerment of caregivers | Home visit observation | Daughter of patient 10 was noted as providing information about the different antibiotic regime, etc. (…), proactively raised concerns about rashes and showed them to the doctor. |
| Patient-provider dynamics in remote and home visits | Importance of home visits | Interview | *“ …ya, home visits make me feel less Kan Cheong (colloquial for anxious), …ya I want them definitely”* – Patient 11 |
|  | Challenges of operating technology for remote visits | Virtual consultation observation | *picture shown by the patient for the medication cannot be seen, had to restart the call by asking the patient to leave and rejoin*– Patient 11 *Healthcare providers seem frustrated due to the poor resolution of the camera used. Ask the patient to use phone to take photos of the medication*– Patient 20 |
|  | Challenges in complex communication in remote visits | Home visit observation | The doctor checked with the patient 19 about the technique and position of leg elevation, which is not something the doctor has normally asked or seen this patient over the video call before. The patient verbalized that she had not known she was doing it wrongly so far, based on what she had understood from the previous virtual consultation |
|  |  | Interview | “*All I remember is* *they told me that this one [HaH care] is 40% cheaper than staying in the ward but now I don’t know what the bill is like…I worried if I hear properly*.” – Patient 6 |
|  |  | Interview | “*things like hospitalization leave, I didn’t actually know how it worked till the doctors told me (…) can be covered with MC (Medical Certificate) to rest at home*.” – Patient 3 |
|  | Barriers and facilitators to rapport building | Home visit observation | Patient 12 and his [vendor] nurse observed as smiling and laughing lightly/chuckling in response to the nurse’s jokes and seemed to have a friendly relationship. This was not evident among all the patient-nurse dyads. |
|  |  | Home visit observation | Patient 10 “seemed to be eager to observe what the doctor was doing and appeared to be on standby to render assistance or information where required” |
|  |  | Virtual consultation observation | The caregiver and the doctor had a direct conversation, and patient 23 appeared to be excluded from the discussion. |
| Complexities of the home environment as a site of care | Home Environment | Home visit observation | Nurses were seen squinting due to dim lighting and shadow, nurses were seen using their phone lights while trying to change their patient’s dressing, and squeezing past (…) a narrow gap by patient 10’s bedside table while trying to access the dressing site. |
|  | Needing advanced notice for home visits | Interview | “I *waited, waited, waited then never come. I also don’t know what happened I also cannot sleep cannot do anything; I don’t know the time they might come what right*.” – Patient 7 |
|  |  | Interview | “I *didn’t know what time they are coming … the last one they came right, I was actually asleep. I was more like shocked.”* – Patient 21 |
|  | Perceived risks of the home environment | Interview | “*scared my illness can affect my other family also*” – Patient 24 |
|  |  | Interview | “…*in a hospital environment there are infection control measures…at home nothing…this is worrying*” – Patient 9 |
|  |  | Interview | “*The last IV drip they sent over. It’s a third-party issue la ... Basically, they just left it, erm. Outside the house … my name, my name is there*.” – Patient 3 |
|  |  | Home Visit Observation | Both patient 9 and the family members appear visibly worried about the infection control measures in their homes. The caregiver was seen making hand gestures towards piles of boxes (which appeared to contain household things) a few times during the visit. |
|  | Hidden Costs | Interview | *“[transition to HaH] so busy… so in the end, I mostly bought all the soup-based [food].”* – Patient 11 |
|  | Role confusion | Interview | *“In [the] hospital definitely the nurse [will not] allow [the patient to do cooking or other household chores] right, but in [the] house ah you [are by] yourself ah so your family say, oh okay she can do everything what, what’s the problem”* – Patient 18 |
|  |  | Interview | *“unofficially, unofficially, I work from home already…bo pian [no choice]...”* – Patient 19 |
|  |  | Virtual Consultation Observation | The caregiver’s laptop with the email page was on and was seen beside the patient's 23 bedside. It appears the caregiver was working from home. |
