# Supplementary material for: Hybrid Hospital-at-Home Program in Singapore: Ethnographic Study
Source: J Med Internet Res. 2025 Jun 2;27:e66107. doi: 10.2196/66107 (PMC12171641; doi:10.2196/66107)
Supplement: Multimedia Appendix 3 [file jmir_v27i1e66107_app3.docx]

**TABLES**

**Table 1.** Demographic profile of participants

| **Characteristics** | **Patients (n = 21)** |
| --- | --- |
| Age (mean, SD)  Sex (*%, n*)  Male  Female  Race/ethnicity (*%, n*)  Chinese  Malay  Indian  Employment (*%, n*)  Employed full-time  Employed part-time  Self-employed  Unemployed/retired  Patient’s primary diagnosis (*%, n*)  Rhabdomyolysis  Dengue  Cellulitis  Acute tonsillitis  Urinary tract infection  Pneumonia  Fluid overload  Fever  Length of stay in HaH program (mean, SD) | 56.7 (24.4)  38.1 (8)  61.9 (13)  47.6 (10)  33.3 (7)  19.0 (4)  42.9 (9)  9.5 (2)  14.3 (3)  33.3 (7)  14.3 (3)  4.8 (1)  42.9 (9)  4.8 (1)  14.3 (3)  9.5 (2)  4.8 (1)  4.8 (1)  5.24 (1.51) |
