# Supplementary material for: Hybrid Hospital-at-Home Program in Singapore: Ethnographic Study
Source: J Med Internet Res. 2025 Jun 2;27:e66107. doi: 10.2196/66107 (PMC12171641; doi:10.2196/66107)
Supplement: Multimedia Appendix 4 [file jmir_v27i1e66107_app4.docx]

**FIGURES**

**Theme 2:**

**Patient-provider dynamics in remote and home visits**

- 1. Importance of home visits
  2. Challenges of operating technology for remote visits
  3. Challenges in complex communication in remote visits
  4. Barriers and facilitators to rapport building

**Theme 3:**

**The complexities of the home environment as a site of care**

- 1. Home environment
  2. Needing advanced notice for home visits
  3. Perceived risks of the home environment
  4. Hidden costs
  5. Role confusion

**Theme 1:**

**Positive experiences of remote and home visits in HaH**

- 1. Feelings of comfort, convenience and safety
  2. Empowerment of patients
  3. Empowerment of caregivers

**Figure 1.** Key themes and subthemes
